# Successful management of a high-risk acute myeloid leukemia patient with severe coronary heart disease by venetoclax plus azacytidine and coronary artery bypass grafting

**DOI:** 10.2478/jtim-2023-0139

**Published:** 2024-03-21

**Authors:** Wen-Jing Yu, Ying Wu, Jian Liu, Yu Chen, Rui-Qin Hou, Xiao-Jun Huang, Hao Jiang

**Affiliations:** Peking University People’s Hospital, Peking University Institute of Hematology, National Clinical Research Center for Hematologic Disease, Beijing Key Laboratory of Hematopoietic Stem Cell Transplantation, Beijing 100044, China; Cardiology Department, Peking University People’s Hospital, Beijing, China; Cardiac Surgery Department, Peking University People’s Hospital, Beijing 100044, China; Department of Transfusion, Peking University People’s Hospital, Beijing 100044, China; Peking-Tsinghua Center for Life Sciences, Beijing 100871, China; Collaborative Innovation Center of Hematology China, Peking University, Beijing 100871, China

## To the editor

Acute myeloid leukemia (AML) mostly occurs in middle-aged and elderly patients, and coronary heart disease (CHD) is one of the most common concomitant diseases in this population.^[1]^ AML patients with severe CHD are extremely challenging for clinicians, moreover, there is no clinical experience to follow for this group of patients, and there is also a lack of relevant literature reported.

**Figure 1:**
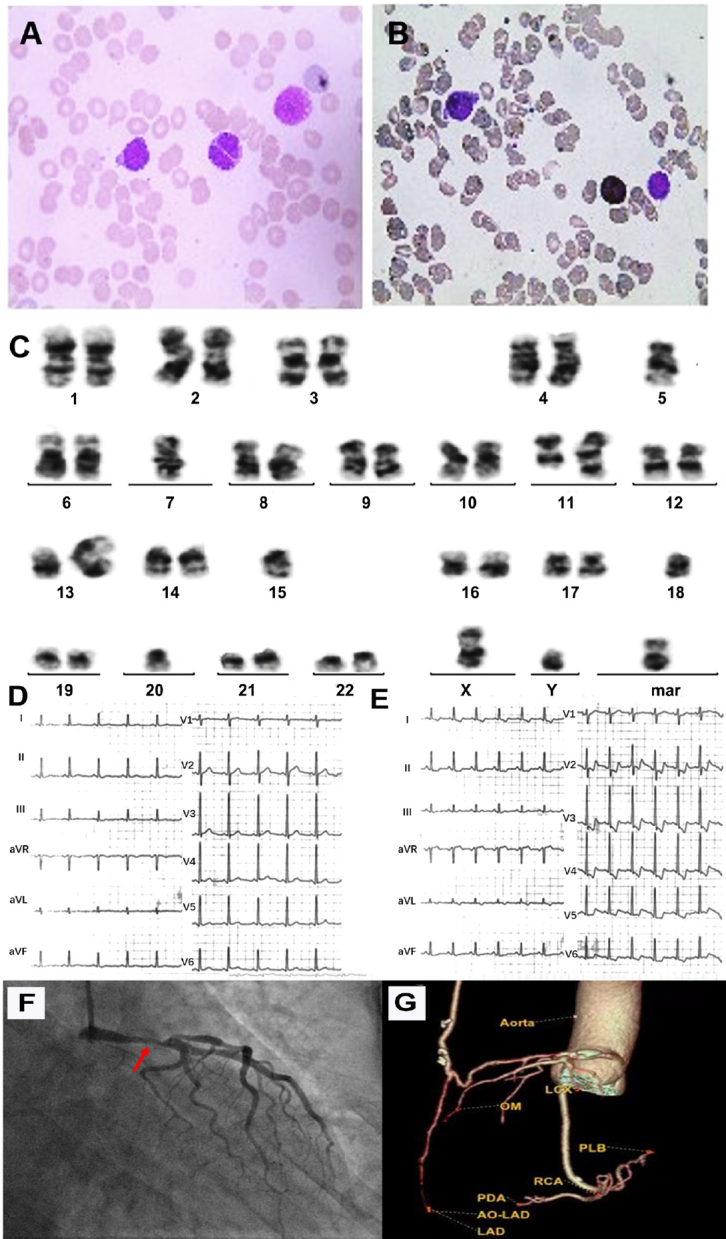
(A) The bone marrow cell morphology of the patient at diagnosis. (B) Peroxidase (POX) staining of bone marrow cells: the cells stained positive for POX. (C) Cytogenetic analysis at diagnosis of the patient: 39-42, XY, add (3)(q21)[2],-5[16],-7[16], inv (9)(q13; q34)[6], +inv (9)(q13q34)[4], dup (11)(q13q23) [6], der (13; 15)(q10; q10)[16],-18 [16],-20 [8], del (20)(q11)[5],-21 [10], +mar1[12], +mar2 [4][cp16] /46, XY[4]. ECG before (D) and after (E) exercise showed that ST segment elevation in aVR lead and ST segment depression in V2-V6 leads after exercise. (F) Coronary angiography showed that the patient had severe stenosis (50%-90% x 15 mm) with plaque in his left main coronary artery. (G) Coronary CT angiography showed that the bypass artery was unobstructed.

A 53-year-old male with bone marrow biopsy was reviewed suggestive possibility of myelodysplastic syndrome, and the gene test showed that TP53 mutation was positive with a complex chromosome karyotype (Figure 1A-C). In addition, the patient had a 2-year history of exertional angina pectoris. electrocardiogram (ECG) after exercise at other hospital showed that ST-segment elevation in lead aVR and ST-segment depression in V2-V6 leads (Figure 1D and 1E). The cardiac ultrasound showed a left-ventricular ejection fraction of 67.9%, without ventricular wall movement abnormality. Chest computed tomography (CT) scan showed no active infections or pleural effusion. The bone marrow morphology in our hospital reported that the patient has rapidly progressed to AML within 2 weeks, with 20.5% myeloblasts.

As the patient was suffering from AML and CHD at the same time, our hospital provided him with multidisciplinary consultation. We collaborated through multi-disciplinary teams (MDT) to develop an overall treatment plan for him. During the first MDT consultation and discussion, according to the opinion of cardiology consultation, the patient was likely to have the problem of the left main coronary artery stenosis. Coronary angiography was performed and the result showed that the patient had severe stenosis with plaque in his left main coronary artery (Figure 1F). Considering the patient was at risk of sudden death due to myocardial infarction at any time, and was almost unlikely to tolerate chemotherapy, the hematology department suggested an immediate intervention of the coronary artery stenosis before the chemotherapy of the high-risk leukemia because the stenosis might be a barrier to chemotherapy. And the percutaneous coronary intervention (PCI) was considered and then rejected because of the strict requirement of continuous dual antiplatelets therapy within at least three months after PCI, which was rather difficult during the chemotherapy or the subsequent hematopoietic stem cell transplantation (HSCT) because of the treatment-related thrombocytopenia. Luckily, the cardiac surgeons were able to provide the patient with the minimally invasive direct coronary artery bypass (MIDCAB), which had advantages of being less invasive, with a shorter recovery time and its postoperative antiplatelet therapy was not must because the bridging vessel is an arterial bridge. Overall, it was decided that the patient would be admitted for MIDCAB as soon as possible before the start of anti-leukemia treatments.^[2]^ However, the patient had high fever on the second day after admission, and the blood culture showed bacteremia, and his complete blood count (CBC) showed pancytopenia. The patient had contraindication to undergo surgery. So, the patient had to be admitted for AML treatment. He received combined anti-infective therapy of linezolid and voriconazole, then the blood culture turned negative.

The patient’s leukemia was classified as high-risk AML.^[3]^ According to the European LeukemiaNet (ELN) guidelines, AML with TP53 mutation and complex karyotype is defined as very adverse. The regimen of Ven and Aza was reported to be safe in patients who were unfit for intensive chemotherapy, and with the composite remission rate of about 55%. What’s more, the regimen has less hematological toxicity than other chemotherapies, so the regimen of Ven and Aza was selected as the induction therapy for this case.^[4,5]^ We conducted bone marrow assessment on the 14^th^ day after initial of chemotherapy, the result showed that myeloblasts had decreased to 8%, and flow cytometry (FCM) showed minimal residual disease (MRD) was negative. Then, on the 21^th^ day of the treatment, the Ven was discontinued due to febrile neutropenia. On the 8^th^ and 11^th^ days after the induction therapy was discontinued, the granulocytes and platelets of the patients began to increase. Worryingly, intermittent chest pain began on the 7^th^ day, and atrial fibrillation occurred on the 10^th^ day after Ven treatment was discontinued. On the 13^th^ day after the Ven was discontinued, the results of CBC gradually recovered to white blood cell (WBC) 2.02 × 10^9^/L, hemoglobin (Hb) 92 g/L, platelet (PLT) 55 × 10^9^/L, and cardiac surgeons decided to perform MIDCAB immediately for him. In the fourth week after the Ven and Aza treatment, the CBC of the patient completely returned to the normal level, and the bone marrow smear examination showed morphological complete remission (CR) and MRD was negative. We performed two cycles of consolidation chemotherapy with the same regimen, and thrombocytopenia did not happen during the subsequent two consolidation therapies, which provided chance for his antiplatelets therapy. The patient received haploidentical HSCT about three months after the surgery. He is doing well at four months after HSCT. In addition, his bypass artery assessed at the cardiac surgery clinic was unobstructed (Figure 1G). However, the patient’s leukemia relapse occurred at the fifth month after HSCT, and finally died of his relapsed leukemia in approximately six months after HSCT. After relapse, the patient received the chemotherapy followed by donor lymphocyte infusion (chemo-DLI), however, he failed to response to the regimen. The patient finally died of severe infection and DIC.

AML with TP53 mutation and complex karyotype is defined as very adverse, and the survival was reported to be very poor. Allo-HSCT could partially improve the survival, however, the long-term survival rate of these patients is still unsatisfactory. Although the patient died of the relapse of the high-risk leukemia, he received the most active regimen to treat his leukemia, including the HSCT and chemo-DLI after relapse. The severe CHD after the MIDCAB did not become an obstacle to his treatment of leukemia. He did not experience any cardiovascular ischemic events throughout the entire treatment process.

This case suggested that patients with the same conditions could benefit from MDT, the efficacy and safety of Ven plus Aza, and optimal opportunity of appropriate surgical intervention.
